# Entropy-Based Patent Valuation: Decoding “Costly Signals” in the Food Industry via a Robust Entropy–TOPSIS Framework

**DOI:** 10.3390/e28020159

**Published:** 2026-01-31

**Authors:** Xiaoman Li, Wei Liu, Xiaohe Liang, Ailian Zhou

**Affiliations:** 1 School of Management, Henan University of Technology, Zhengzhou 450001, China; lixiaoman@haut.edu.cn (X.L.); liuwei@haut.edu.cn (W.L.); 2 Institute of Agricultural Information, Chinese Academy of Agricultural Sciences, Beijing 100081, China

**Keywords:** Entropy–TOPSIS, patent valuation, food industry, costly signaling, heavy-tailed data

## Abstract

Accurate patent valuation remains a persistent challenge in intellectual property management, particularly in the food industry, where technological homogeneity and rapid innovation cycles introduce substantial noise into observable performance indicators. Traditional valuation approaches, whether based on subjective expert judgment or citation-based metrics, often struggle to effectively reduce information uncertainty in this context. To address this limitation, this study proposes an objective, data-driven patent valuation framework grounded in information theory. We construct a multidimensional evaluation system comprising nine indicators across technological, legal, and economic dimensions and apply it to a large-scale dataset of 100,648 invention patents. To address the heavy-tailed nature of patent indicators without sacrificing the information contained in high-impact outliers, we introduce a square-root transformation strategy that stabilizes dispersion while preserving ordinal relationships. Indicator weights are determined objectively via Shannon entropy, capturing the relative scarcity and discriminatory information content of each signal, after which comprehensive value scores are derived using the TOPSIS method. Empirical results reveal that the entropy-based model assigns dominant weights to so-called “costly signals”, specifically PCT applications (29.53%) and patent transfers (24.36%). Statistical correlation analysis confirms that these selected indicators are significantly associated with patent value (p<0.001), while bootstrapping tests demonstrate the robustness of the resulting weight structure. The model’s validity is further evaluated using an external benchmark (“ground truth”) dataset comprising 55 patents recognized by the China Patent Award. The proposed framework demonstrates substantially stronger discriminatory capability than baseline methods, awarded patents achieve an average score 2.64 times higher than that of ordinary patents, and the enrichment factor for award-winning patents within the Top-100 ranking reaches 91.5. Additional robustness analyses, including benchmarking against the Weighted Sum Model (WSM), further confirm the methodological stability of the framework, with sensitivity analysis revealing an exceptional enrichment factor of 183.1 for the Top-50 patents. These findings confirm that the Entropy–TOPSIS framework functions as an effective information-filtering mechanism, amplifying high-value patent signals in noise-intensive environments. Consequently, the proposed model serves as a generalizable and theoretically grounded tool for objective patent valuation, with particular relevance to industries characterized by heavy-tailed data and high information uncertainty.

## 1. Introduction

Innovation in the food industry is undergoing a structural transformation, shifting from traditional manufacturing practices toward technologically intensive domains such as functional foods, bio-manufacturing, and precision nutrition. In this evolving landscape, patents have become a core intangible asset enabling firms to secure competitive advantage. However, the rapid expansion in patent filings has produced a well-recognized “patent paradox,” whereby quantitative growth does not necessarily translate into qualitative improvement in innovation output [1]. Consequently, identifying high-value patents those possessing significant technological merit and market potential from a vast ocean of “sleeping patents” has become a pressing challenge for policymakers, investors and corporate managers [2].

Although numerous patent valuation approaches have been proposed in academia and practice, their application to the food industry remains limited by sector-specific characteristics. Unlike industries such as pharmaceuticals or information and communication technologies (ICT), food-related innovations are typically incremental and process-oriented, while firms operate under strong cost constraints [3]. This results in poor discrimination power when using general evaluation indicators like raw citation counts, which often fail to distinguish subtle value differences in highly homogenized patent data. Furthermore, existing evaluation systems often face a dilemma: subjective expert assessments (e.g., the Delphi method [4], while authoritative, are constrained by cognitive biases, high costs, and an inability to process massive datasets; conversely, traditional quantitative analyses often rely on citation data, which suffer from severe “time lag” effects and struggle to extract key value signals from highly homogenized food patent data [5]. Fundamentally, the objective quantification of patent value from noisy big data is a process of “reducing information uncertainty”.

To address these challenges, this study introduces Information Entropy theory into the domain of patent valuation.In multiple-criteria decision-making (MCDM), the entropy-weight method determines indicator weights solely according to the dispersion of data [6]. Indicators exhibiting higher variation and thus lower entropy carry more discriminatory information and therefore receive higher weights. This property makes the method particularly suitable for capturing “sparse signals” such as PCT applications and patent transfers, which occur infrequently but convey strong information regarding technological and commercial value.

Building on this signal-detection perspective, this study makes three primary contributions to the patent valuation literature. First, it develops an objective entropy–TOPSIS framework that conceptualizes patent valuation as an information-filtering process, enabling the amplification of scarce but high-information commercial and legal signals under noise-intensive and heavy-tailed data environments. Second, by incorporating square-root data transformation and entropy-based weighting, the proposed approach mitigates the distortion effects of extreme outliers while preserving ordinal information critical for identifying high-value patents. Third, the study provides rigorous empirical validation using awarded patents as an independent external ground truth, thereby overcoming the circular logic commonly found in indicator-based valuation models. Together, these contributions offer a theoretically grounded and practically robust solution for micro-level patent valuation in the food industry.

## 2. Literature Review

### 2.1. Multidimensional Construction of Patent Value Indicators

Patent value is a latent variable that cannot be directly observed, which necessitates the construction of a multidimensional proxy framework [7]. Contemporary literature typically decomposes patent value into technological, legal, and economic dimensions to capture its multifaceted nature [5].

At the indicator design level, the technical dimension measures the intrinsic innovation quality, covers core indicators such as patent forward citations, number of IPC classifications, inventor counts, and Technology Life Cycle (TLC), reflecting technological advancement, coverage scope, R&D investment intensity and maturity [8]. The legal dimension evaluates the scope of protection, stability and regional monopolistic capacity through indicators including number of claims, patent family size, patent lifespan and number of litigation cases [9]. The economic & market dimension focuses on commercialization potential indicators like number of patent transfers, patent licensing and transfer family size (reflecting market layout) and maintenance duration, which reflect market recognition and economic lifespan while quantifying commercialization potential [10]. The strategic dimension is associated with indicators including the number of emerging industries, the number of green industries, the number of knowledge-intensive industries, the number of core digital economy industries, the duration of public availability, cooperation status overseas layout, disciplinary support, the number of patent invalidation claims against the subject, patent award records, and strategic emerging industries, revealing the potential of technological restructuring and policy compatibility [11,12,13].

However, the food industry differs significantly from high-tech sectors. Research by Yoshioka-Kobayashi et al. (2020) [3] shows that innovation in the food sector is predominantly incremental and process-oriented, unlike the discrete product innovation seen in pharmaceuticals. Furthermore, a review by Bigliardi and Filippelli (2022) emphasizes that the agro-food industry is traditionally characterized as a “low-tech” sector with low R&D intensity, where firms are highly sensitive to production costs and market fluctuations [14]. Consequently, general-purpose indicators like raw citation counts often exhibit limited discriminatory power in this homogenized field, which necessitates a valuation framework that prioritizes “costly signals” like international filings over routine technical metrics.

### 2.2. Methodological Evolution: From Subjective Scoring to Intelligent Computation

The paradigm for patent valuation has evolved from qualitative expert judgment to quantitative data-driven analytics [4]. Early multi-criteria decision-making methods, such as the Analytic Hierarchy Process (AHP) and the Delphi method relied heavily on expert experience for indicator screening and weight assignment [4]. While these methods provide intuitive interpretability for small samples,, they also suffer from high subjectivity, poor robustness, and an inability to scale to massive datasets.

In recent years, machine learning (ML) and deep learning have been extensively introduced to enhance evaluation efficiency. For example, Kayakökü and Tüfekci (2025) developed a Siamese neural network based model to detect patentability through semantic similarity analysis [15]. Jing et al. (2025) developed a multimodal approach combining patent knowledge graphs and image similarity to predict infringement risks [16]. Additionally, Gao et al. (2025) introduced a Bayesian-optimized XGBoost model, which significantly improved prediction accuracy and operational efficiency compared to traditional models [17]. Along this research stream, an increasing number of studies have begun to integrate deep learning with textual information, citation or network embeddings, and ontology-based representations to support patent prediction, valuation, and recommendation tasks, forming an emerging paradigm in data-driven patent analytics [18,19,20].

Beyond pure prediction, configuration analysis has gained attention. Wang et al. (2024) utilized Fuzzy-Set Qualitative Comparative Analysis (fsQCA) on Chinese Patent Award winners, revealing that high value is not driven by a single factor but by specific “configuration paths” of technological, legal, and economic attributes [21]. Li et al. (2023) developed a multidimensional patent-indicator pre-screening strategy combined with machine-learning models to evaluate and identify potential high-value patents in the integrated-circuit domain, providing a representative empirical framework for data-driven patent value assessment [2].

Despite these advancements, ML and deep learning models often function as “black boxes,” lacking the logical transparency required for decision making. This limitation has stimulated renewed interest in transparent analytical frameworks that balance objectivity with interpretability.

### 2.3. Application of Information Entropy in Evaluation

To balance objectivity with logical transparency, the Entropy Weight Method (EWM) based on Shannon’s information theory has garnered increasing attention [22,23]. In information theory, entropy quantifies the uncertainty of a signal source. When applied to multi-criteria decision-making, EWM determines weights based on the dispersion of data: an indicator with high variability (low entropy) carries more discriminatory information and is thus assigned a higher weight. This follows the objective logic that “the greater the variation, the richer the information,” effectively avoiding the bias of human weighting [6].

Recent empirical studies have validated the robustness of the Entropy–TOPSIS framework in objective technology assessment; for instance, Yuan and Song (2022) successfully applied this hybrid model to evaluate the innovation capabilities of solar cell companies, demonstrating its superiority in mitigating subjective bias and handling multi-dimensional data [24]. However, current applications of entropy in patent analysis are predominantly focused on macro-level evaluations, such as evaluating regional or industrial innovation performance [22]. Research focusing on micro-level like targeting the valuation of individual patents remains comparatively scarce [4]. Furthermore, patent data typically follow a heavy-tailed, power-law distributions. Few studies have systematically examined how entropy weighting interacts with with data transformation techniques to mitigate the weight distortion caused by extreme outliers in the long tail.

### 2.4. Research Gaps and the “Signal Detection” Perspective

Despite the richness of existing literature, two critical methodological gaps remain, providing the impetus for this study. First, existing studies often lack rigorous external validation based on authoritative ground-truth benchmarks. Most research defines high-value patents recursively using internal indicators, such as high citation counts or composite scores derived from the same variables employed in model construction, thereby introducing circular logic and methodological endogeneity. Empirical validation against independent and widely recognized benchmarks, such as patent award systems, remains scarce in the patent valuation literature.

Second, existing studies insufficiently explore the role of “costly signals.” In the food industry, indicators such as PCT international applications and patent transfers represent high-cost strategic actions that signal strong commercial confidence. However, these indicators often appear as sparse binary variables and are easily overshadowed by continuous variables in linear models. Current literature has not fully examined how entropy weighting may amplify the informational content of these rare but economically meaningful signals.

Third, although information entropy has been increasingly applied to innovation and technology evaluation, its use in patent analysis remains largely confined to macro-level assessments, such as regional or industrial innovation performance. Micro-level valuation focusing on individual patents, particularly under heavy-tailed data distributions, remains underexplored. Moreover, few studies have systematically examined how entropy-based weighting interacts with data transformation strategies to mitigate weight distortion caused by extreme outliers in long-tailed patent data.

In response to these gaps, this study adopts a signal-detection perspective and proposes an objective patent valuation framework integrating Shannon entropy and the TOPSIS method. By employing awarded patents as an independent external ground truth and explicitly addressing heavy-tailed data characteristics, this research aims to demonstrate the unique effectiveness of entropy-based weighting in amplifying scarce but high-information commercial and legal signals within noise-intensive food industry patent systems.

## 3. Methodology and Model Construction

### 3.1. Construction of the Patent Value Evaluation System

#### Dimensions and Indicator Selection

Based on existing patent evaluation frameworks and the specific industrial attributes of the food sector, this study constructs a hierarchical evaluation system comprising nine core indicators across three dimensions: Technological, Legal, and Market & Economic. This multidimensional system enables the measurement of technological merit, legal enforceability, and commercial attractiveness in an integrated framework consistent with the economic nature of patent value.

The technological dimension reflects technical complexity, protection breadth, and R&D commitment. Number of Claims ($X_{1}$): This indicator captures the granularity and breadth of patent protection. A larger number of claims generally indicates more sophisticated technical content and a broader exclusion scope. Examination Duration ($X_{2}$): In this study, examination duration is treated as a positive indicator. Food biotechnology patents involving complex biochemical processes often undergo extended substantive examination, which may signal higher technical complexity and strategic importance. IPC Count ($X_{3}$): The number of IPC subclasses reflects the degree of technological diversification and interdisciplinary application potential.

The legal dimension captures applicants’ willingness and capability to maintain and protect patent rights. Legal Event Occurrence ($X_{4}$): This indicator records legal activity such as transfers, pledges, licensing, and litigation. Frequent legal events typically reflect active asset management rather than passive right-holding. PCT Application ($X_{5}$): PCT filing serves as a strong signal of internationalization. Given the relatively thin profit margins in the food industry, a PCT application constitutes a “costly signal,” implying strong commercial expectations.

The market & economic dimension represents commercialization potential and industrial influence.Forward Citations ($X_{6}$): This measures knowledge diffusion and technological spillover intensity. A higher citation count places the technology at the upstream core of the industry’s R&D chain. Patent Transfer ($X_{7}$): Transfer records provide direct evidence of market recognition and asset monetization. A transfer record implies that the technology has completed value discovery and ownership exchange through market mechanisms, confirming its economic value. Number of Applicants ($X_{8}$): Joint applications often imply a background of Industry University Research cooperation, usually corresponding to technologies with higher practical applicability. Emerging Industry Classification ($X_{9}$): This reflects alignment with national strategic emerging industries, capturing policy relevance and future growth potential.

Patent data were retrieved from the PatSnap global patent database. The search strategy focused on invention patents published between 2018 and 2019 within specific IPC subclasses relevant to the food industry, including A21D (flour and dough), A23B (preservation), A23C (dairy products), A23D (oils and fats), A23F (coffee and tea), A23G (cocoa and confectionery), A23J (proteins), and A23L (general foods).The query was defined as: MIPC:(A21D OR A23B OR A23C OR A23D OR A23F OR A23G OR A23J OR A23L) AND PY = (2018–2019).

After data cleaning to remove records with invalid legal status or missing critical fields, a final dataset of 100,648 valid invention patents was obtained. To verify the objectivity and accuracy of the evaluation model, we matched 55 patents within this domain that had won the China Patent Award (Gold, Silver, and Excellence Awards) to serve as the “Ground Truth” dataset.

### 3.2. Entropy–TOPSIS Patent Value Measurement Model

#### 3.2.1. Data Preprocessing: Stabilizing Heavy-Tailed Distributions

Patent indicators, particularly forward citations and family sizes, typically follow heavy-tailed, power-law distributions. Extreme outliers may dominate Euclidean-distance calculations in TOPSIS and distort entropy-based weights, potentially causing model instability.

**Figure 1:**
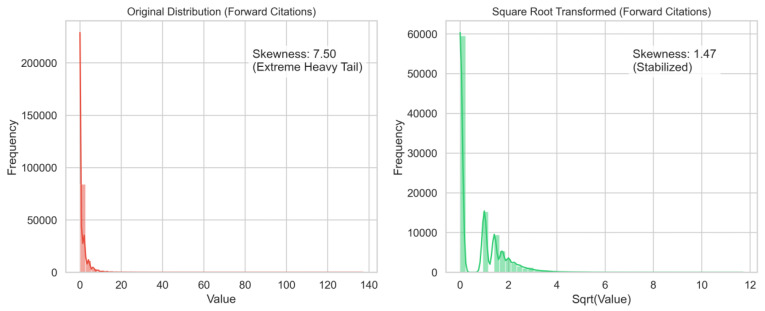
Comparison of Data Distribution Before and After Square Root Transformation.

To mitigate this issue, all continuous variables were subjected to a square-root transformation. The effectiveness of this approach was empirically assessed by comparing the statistical dispersion of key indicators before and after transformation. As shown in Figure 1, the transformation substantially reduced the heavy-tailed characteristics of the data; for instance, the skewness of forward citations decreased from 7.50 (raw) to 1.47 (transformed). This adjustment moves the distribution closer to normality while suppressing the influence of extreme outliers, without the information loss associated with truncation methods such as winterization.

Following this transformation, all indicators were normalized to the [0, 1] interval using Min-Max scaling to eliminate dimensional differences:

(1) $$x_{ij}^{\prime }=\frac{x_{ij}-\min\left(x_{j}\right)}{\max\left(x_{j}\right)-m\min\left(x_{j}\right)},$$

where $x_{ij}$ is the transformed value of the *j*-th indicator for the *i*-th patent.

#### 3.2.2. Objective Weighting Based on Information Entropy

To eliminate subjectivity in indicator weighting, the entropy weight method (EWM) grounded in Shannon’s information theory was employed. In this framework, entropy quantifies the uncertainty of a signal, lower entropy indicates greater dispersion and, consequently, higher discriminatory information content (weight).

This characteristic renders the entropy method particularly suitable for the food industry, where technological homogeneity prevails. For uniform indicators such as claim counts, where most patents display similar values, the method assigns low weights due to low variance, effectively filtering out “noise”. Conversely, for sparse, high-cost actions such as PCT applications, higher variance leads to greater weights. Therefore, the entropy method operates as an objective “signal amplifier” for rare but high-information events.

- Step 1: Calculate the probability proportion $\left(p_{ij}\right)$:
  (2) $$p_{ij}=\frac{x_{ij}^{\prime }}{\sum_{i=1}^{m}x_{ij}^{\prime }}$$
- Step2: Calculate the Information Entropy $\left(e_{j}\right)$:
  (3) $$e_{j}=-k\sum_{i=1}^{m}p_{ij}\ln p_{ij},$$
  where *m* is the sample size, and k=1/(ln(m)) is the normalization constant.
  (4) $$\mathrm{If}\;p_{ij}=0,\mathrm{define}\;p_{ij}\ln\left(p_{ij}\right)=0.$$
- Step 3: Determine Entropy Weight $\left(w_{j}\right)$:
  (5) $$w_{j}=\frac{1-e_{j}}{\sum_{j=1}^{n}\left(1-e_{j}\right)}$$
  where $1-e_{j}$ represents the coefficient of variation, *n* is the number of indicators, ensuring $\sum_{j=1}^{n}w_{j}=1$.

#### 3.2.3. Comprehensive Evaluation via TOPSIS

The TOPSIS (Technique for Order Preference by Similarity to Ideal Solution) method ranks patents by measuring their geometric distance to the ideal state. This model ranks patents by calculating the geometric distance to the “Positive Ideal Solution” ($Z^{+}$) and the “Negative Ideal Solution” ($Z^{-}$).

The weighted normalized matrix is obtained as:

(6) $$z_{ij}=w_{j}\times x_{ij}^{\prime }$$

The positive ideal solution (PIS) and negative ideal solution (NIS) are defined as:

$$\begin{array}{cc} \; & \mathrm{Positive}\;\mathrm{Ideal}\;\mathrm{Solution}\;(Z^{+}):\mathrm{The}\;\mathrm{vector}\;\mathrm{of}\;\mathrm{maximum}\;\mathrm{values}\;\mathrm{for}\;\mathrm{each}\;\mathrm{indicator}. \end{array}$$

(7) $$Z^{+}=\left(max\left(z_{i1}\right),max\left(z_{i2}\right),\ldots ,max\left(z_{in}\right)\right)$$

$$\begin{array}{cc} \; & \mathrm{Negative}\;\mathrm{Ideal}\;\mathrm{Solution}\;(Z^{-}):\mathrm{The}\;\mathrm{vector}\;\mathrm{of}\;\mathrm{minimum}\;\mathrm{values}\;\mathrm{for}\;\mathrm{each}\;\mathrm{indicator}. \end{array}$$

(8) $$Z^{-}=\left(min\left(z_{i1}\right),min\left(z_{i2}\right),\ldots ,min\left(z_{in}\right)\right)$$

The Euclidean distances between each patent and the two ideal solutions are computed as:

(9) $$D_{i}^{+}=\sqrt{\sum_{j=1}^{n}{\left(z_{ij}-Z_{j}^{+}\right)}^{2}},D_{i}^{-}=\sqrt{\sum_{j=1}^{n}{\left(z_{ij}-Z_{j}^{-}\right)}^{2}}$$

Finally, the relative closeness to the ideal solution is expressed as:

(10) $$S_{i}=\frac{D_{i}^{-}}{D_{i}^{+}+D_{i}^{-}}$$

The score $S_{i}$ ranges from [0,1]. A higher value indicates the patent is closer to the ideal solution and farther from the negative ideal.

### 3.3. Model Validation Strategy

Unlike traditional machine learning approaches that split data into training and testing sets, this study employs a hypothesis testing framework using external “Ground Truth” data.

**(1) Statistical Significance Test**

We categorize the sample into an “Awarded Group” (N=55) and an “Ordinary Group” (N=100,648). The non-parametric Mann-Whitney U test is used to determine if the comprehensive scores of the Awarded Group are statistically significantly higher than those of the Ordinary Group (p<0.05).

**(2) List Enrichment Analysis**

To measure the model’s efficiency in “mining” value from noise, we calculate the Enrichment Factor in the top-ranked patents:

(11) $$EF_{K}=\frac{\mathrm{Proportion}\;\mathrm{of}\;\mathrm{Awarded}\;\mathrm{Patents}\;\mathrm{in}\;\mathrm{Top}\;K}{\mathrm{Proportion}\;\mathrm{of}\;\mathrm{Awarded}\;\mathrm{Patents}\;\mathrm{in}\;\mathrm{Total}\;\mathrm{Sample}}$$

A high enrichment factor indicates effective signal amplification and noise reduction.

## 4. Empirical Analysis

### 4.1. Descriptive Statistics and Empirical Justification

#### 4.1.1. Descriptive Statistics

To provide an intuitive overview of the distributional characteristics of the dataset, we calculated the descriptive statistics for the Awarded Patent Group (N=55, “Ground Truth”) and the Ordinary Patent Group (N=100,648, “Background Noise”) based on the original raw data. As presented in Table 1, significant disparities are observed across key dimensions.

A pronounced divergence is evident in the legal & market dimensions. The mean value for “Legal Event Occurrence” in the Awarded Group is 0.51, approximately 5.1 times higher than that of the Ordinary Group. Likewise, the incidence rates of “PCT Flag” and “Transfer Flag” in the Awarded Group are roughly three times higher than in the Ordinary Group. These findings indicate that high-value patents in the food industry are systematically associated with stronger signals of active rights operation, market transactions, and international deployment, supporting the relevance of costly legal and commercial behaviors in patent valuation.

Technological indicators also show meaningful differences. Awarded patents exhibit significantly longer “Examination Duration” and higher “Applicants Count”. This suggests that high-value patents are typically linked to greater technological complexity requiring intensive substantive examination and often emerge from collaborative R&D environments. In addition, traditional quality indicators such as Forward Citations and Claims Count remain consistently higher among awarded patents, confirming the basic validity of the indicator system employed in this study.

#### 4.1.2. Empirical Justification of Indicators

**Figure 2:**
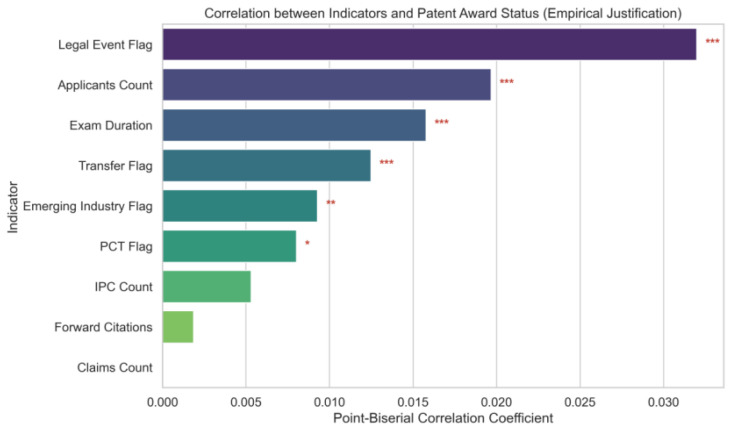
Point-Biserial Correlation between Evaluation Indicators and Patent Award Status (* p<0.05, ** p<0.01, *** p<0.001).

To empirically justify the inclusion of each indicator prior to model construction, a point-biserial correlation analysis was conducted between the transformed indicator values and patent award status. As illustrated in Figure 2, despite the sparsity and noise inherent in patent data, most indicators exhibit statistically significant positive correlations with high-value outcomes.

First, indicators representing “costly signals” and collaborative engagement demonstrate strong explanatory power. Specifically, Legal Event Flag and Applicants Count exhibit strong statistical significance. Notably, Examination Duration also displays a highly significant positive correlation with award status. This evidence supports the interpretation that longer examination cycles in the food industry are more likely to reflect substantive scrutiny of complex technologies rather than administrative delay, thereby empirically justifying its treatment as a positive indicator within the proposed valuation framework.

**Figure 3:**
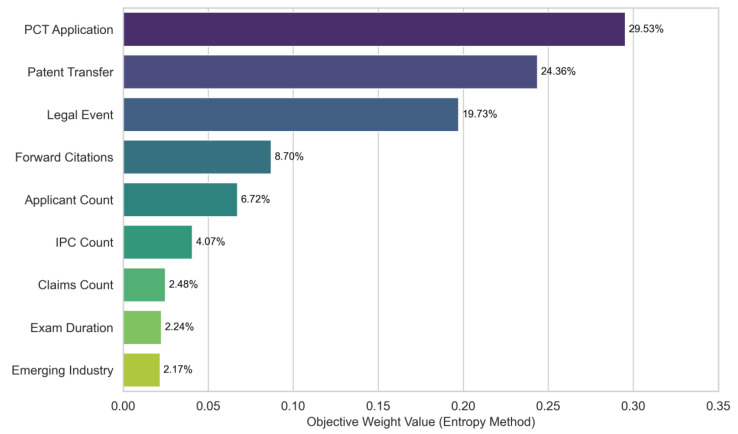
Distribution of Objective Weights for Patent Valuation Indicators based on Information Entropy.

Equally important, several traditional quantitative indicators, including Forward Citations, Claims Count, and IPC Count, do not exhibit statistical significance in this analysis. This result is non-trivial, as it empirically corroborates the “patent paradox” in the food industry discussed in Section 2.1. Given the incremental nature and technological homogeneity of food innovations, these routine metrics often lack sufficient discriminatory power to distinguish high-value assets from ordinary ones. However, rather than excluding these indicators, they are deliberately retained to preserve the theoretical completeness of the multidimensional evaluation framework. Their inclusion further serves to validate the effectiveness of the proposed entropy-based weighting mechanism. As demonstrated in the subsequent weight distribution analysis (Figure 3), the model objectively detects the low dispersion of these indicators and automatically assigns them lower weights. This result highlights the model’s capability to function as an objective information filter, suppressing noise from homogenized metrics while amplifying high-information “costly signals”, without the need for manual intervention.

### 4.2. Entropy-Based Weight Distribution Analysis

The objective weights for each indicator, calculated via the Entropy Weight Method (EWM), are presented in Figure 3. The results present a compelling information-theoretic narrative regarding value definition in the food industry. The analysis identifies three dominant indicators with the highest entropy-based weights: “PCT Application” (29.53%), “Patent Transfer” (24.35%), and “Legal Event Occurrence” (19.73%)—receive the highest entropy-based weights, together accounting for 73.62% of total weight.

According to Shannon’s information theory, the information content of a signal is inversely related to its probability of occurrence. In the food industry, low-cost domestic patent filings are the norm, carrying low discriminatory information. In contrast, PCT applications and market transfers are rare events that require substantial financial commitment. Within the entropy-weighting framework, these sparse and high-cost events exhibit greater dispersion and therefore receive higher weights. They function as effective “costly signals”, enabling the model to distinguish high-value core patents from the homogenized background noise prevalent in the food sector.

By contrast, indicators such as “Emerging Industry Classification” (2.17%) and “Claims Count” (2.48%) receive relatively lower weights. It is important to clarify that this does not negate their theoretical importance; rather, it indicates that their discriminatory power is low within this specific dataset. As discussed in Section 2.1, the food industry is characterized by technological homogeneity, meaning that most patents, regardless of their actual value, tend to possess similar claim structures and industry classifications, resulting in high entropy (uncertainty) and consequently low weighting.

**Figure 4:**
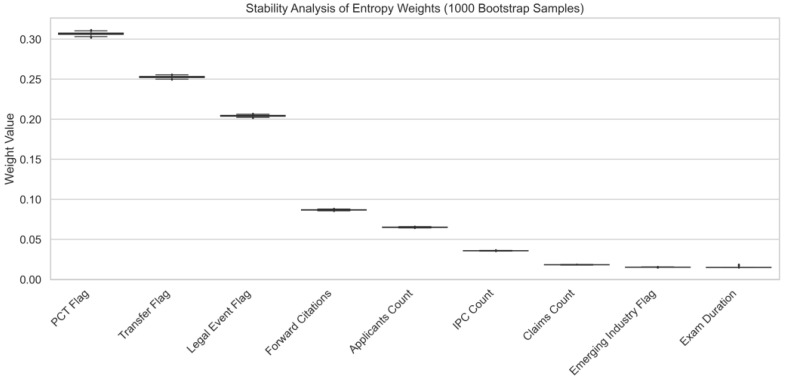
Stability Analysis of Entropy Weights based on 1000 Bootstrap Samples.

To verify that the dominant weights assigned to costly signals (PCT and Transfer) are not artifacts of sampling bias, a bootstrapping-based stability analysis was conducted. Specifically, 1000 bootstrap samples were generated, each containing 80% of the original dataset drawn with replacement, and entropy weights were recalculated for each iteration. As illustrated in Figure 4, the resulting confidence intervals for all indicators are extremely narrow. Notably, PCT Application and Patent Transfer consistently remain the top-weighted indicators across resamples, exhibiting minimal variance. This evidence confirms that the high weights assigned to these indicators reflect their intrinsic information entropy, driven by scarcity and dispersion, rather than random data fluctuations or sampling instability.

### 4.3. Comprehensive Evaluation Results and Model Validation

To rigorously assess the effectiveness of the proposed Entropy–TOPSIS framework in uncovering “true value” from large-scale patent data, we adopted a dual validation strategy that combines statistical distinction testing with ranking-based enrichment analysis.

#### 4.3.1. Statistical Distinction (Mann-Whitney U Test)

**Figure 5:**
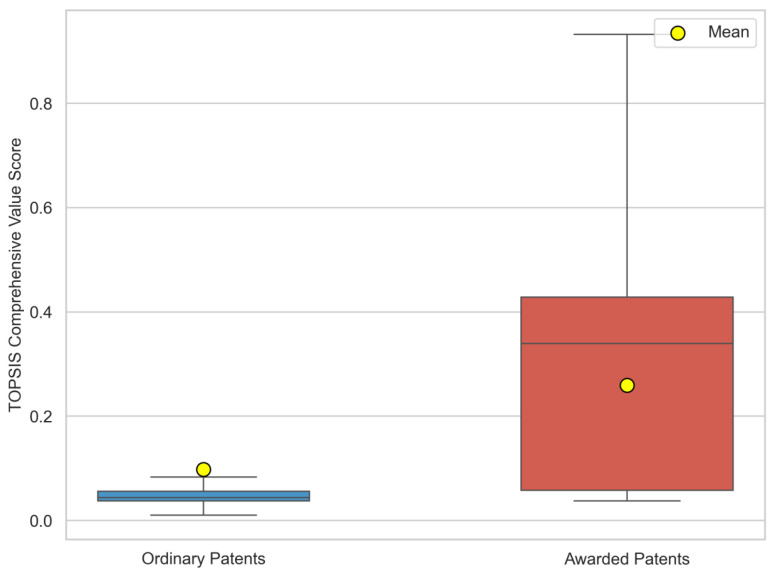
Distribution Comparison of Comprehensive Value Scores between Awarded and Ordinary Patent Groups.

The TOPSIS results show a clear separation between awarded and ordinary patents. As illustrated in Figure 5, the mean comprehensive score of the Awarded Group is 0.2775, which is 2.64 times higher than that of the Ordinary Group. A non-parametric Mann–Whitney U test confirmed that this difference is statistically significant (p<0.001), rejecting the null hypothesis that the score distributions are identical. These findings confirm that the entropy–TOPSIS model successfully maps the multidimensional attributes of “value” onto a separable one-dimensional score space.

#### 4.3.2. Enrichment Factor and Sensitivity Analysis

While statistical separation confirms discriminative validity, practical patent valuation tasks further require the model to efficiently surface rare high-value assets from massive datasets. To evaluate this capability, we conducted an Enrichment Factor (EF) analysis across multiple ranking cutoffs. In the full dataset, awarded patents constitute only 0.055% of all observations, reflecting the extreme rarity of objectively recognized high-value patents. However, among the Top-100 patents ranked by the proposed model, 5 patents are award-winning, corresponding to a proportion of 5.00%. The resulting Enrichment Factor reaches 91.5, indicating that the density of high-value patents in the model-selected Top-100 set is 91.5 times higher than that expected under random selection.

**Figure 6:**
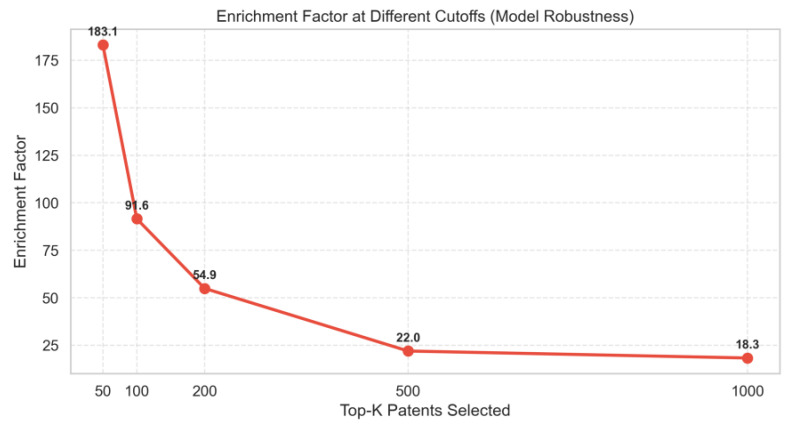
Enrichment Factor at Different Cutoffs (Model Robustness).

To demonstrate the robustness of the ranking, we performed a sensitivity analysis across five different cutoffs (Top-50 to Top-1000). As shown in Figure 6, the Enrichment Factor displays a decreasing trend as the cutoff increases, which is characteristic of an effective ranking system that prioritizes the most valuable assets at the very top. For Top-50, the Enrichment Factor peaks at 183.1, indicating exceptional precision at the top of the list. For Top-500, the factor stabilizes at 22.0, meaning the model still identifies high-value patents with a density 22 times higher than random selection even at a broader scale.This trend confirms the model’s robustness in identifying “hidden champions” at different granularity levels.

### 4.4. Comparative Analysis

To further examine the robustness and methodological validity of the proposed framework, we conducted a two-stage comparative analysis. The first stage focuses on an internal comparison of alternative data transformation strategies within the TOPSIS framework, while the second stage benchmarks the proposed approach against a non-TOPSIS ranking method to assess algorithmic dependence.

#### 4.4.1. Comparison of Data Transformation Strategies

To identify the most appropriate data processing strategy for patent valuation under heavy-tailed distributions, the proposed Square-Root Entropy–TOPSIS model (Model IV) was benchmarked against three alternative models representing distinct methodological assumptions. These include Equal-Weight TOPSIS (Model I), which assumes uniform contribution from all indicators; Raw Entropy–TOPSIS (Model II), which applies entropy weighting on the original dataset without data transformation; Winsorized Entropy–TOPSIS (Model III), which applies a 1% bilateral truncation to suppress extreme outliers.

To quantify the discrimination power of each model, we employed three key evaluation metrics. First, the Differentiation Ratio is defined as the ratio of the mean comprehensive score of the Awarded Group to that of the Ordinary Group, where a higher ratio generally indicates stronger separation between high-value and average patents. Second, the Top-100 Enrichment Factor represents the density of awarded patents within the top 100 ranked results, reflecting the model’s precision in “mining” core assets from the noise. Third, Statistical Significance, based on the Mann-Whitney U test, evaluates whether the two score distributions are statistically distinguishable.

The comparative results in Table 2 reveal critical insights into the mechanism of patent valuation.

(1) The Limitations of equal weighting (Model I vs. Others). Model I exhibits the weakest performance across all metrics. With a differentiation ratio of only 1.60 and an Enrichment Factor of 18.3 (identifying significantly fewer core assets), it confirms that treating common indicators (e.g., claims count) and sparse signals (e.g., PCT applications) equally severely dilutes the critical information contained in “costly signals.” This leads to a low signal-to-noise ratio, failing to effectively highlight high-value patents even when data is properly transformed.

(2) The “Sensitivity Trap” of Raw Data (Model II vs. Model III). An intriguing phenomenon is observed where the Raw Data Model (Model II) achieves the highest numerical differentiation ratio (2.93). While a higher ratio typically suggests better separation, a deeper methodological analysis reveals that this advantage stems from a “Sensitivity Trap.”Raw data assumes a linear value function, implying that a patent with extreme outliers (e.g., hundreds of forward citations) is proportionally more valuable. This allows outliers to dominate the Euclidean distance calculation in TOPSIS, artificially inflating the scores of top patents. While this yields a high separation score, it renders the model fragile to data noise and inconsistencies, potentially overfitting to numerical magnitude rather than systematic value signals.

(3) The Signal loss under truncation (Model III). Winsorization attempts to mitigate outlier interference by truncating the top 1% of data. However, this results in a significant drop in the differentiation ratio to 2.20. This decline indicates that in the context of patent valuation, the top 1% of data points are not merely “noise” but often represent “Super-Star” assets. Truncating them equates to signal loss, artificially capping the advantage of the most groundbreaking innovations.

(4) The optimal balance achieved by the proposed framework (Model IV). The proposed Model IV (Square Root Transformation) achieves the optimal balance. By applying a non-linear transformation ($\sqrt{x}$), it mildly compresses the scale of extreme values without imposing an artificial ceiling. Consequently, it recovers the discriminatory power lost in Model III (rising from 2.20 to 2.64) while avoiding the instability of Model II. The P-value of Model IV is also superior to that of Model III, confirming that the square root transformation provides the most statistically robust representation of patent value in heavy-tailed distributions.

#### 4.4.2. Cross-Validation with Non-TOPSIS Benchmark

**Figure 7:**
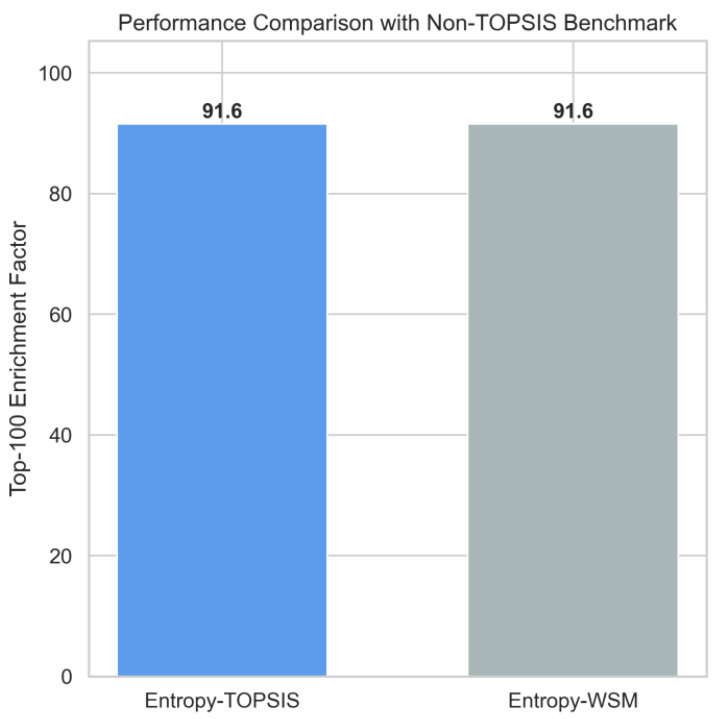
Performance Comparison with Non-TOPSIS Benchmark (Entropy-WSM).

To ensure that the observed performance is not driven by the specific geometric properties of the TOPSIS algorithm, we further benchmarked the proposed framework against the Weighted Sum Model (WSM) using the same entropy-derived weights. As shown in Figure 7, both methods achieve an identical Top-100 Enrichment Factor of 91.6. This consistency indicates that the primary source of performance improvement lies in the entropy-based identification of high-information “costly signals”, rather than in the choice of ranking algorithm itself. The cross-validation thus demonstrates that the proposed valuation framework is algorithm-agnostic and exhibits strong generalization.

### 4.5. Characterization of High-Value Patents

To further interpret the economic and technological meaning of the model outputs, we analyzed the Top-1000 patents identified by the proposed framework to characterize the structural and thematic features of high-value assets in the food industry. This analysis aims to move beyond validation and reveal what kinds of patents are systematically highlighted when value is defined through information entropy and costly signals.

#### 4.5.1. Structural Profiling

High-value patents are heavily concentrated in IPC subclasses A23L (General Foods), A23G (Confectionery), and A23C (Dairy Products). This suggests that recent innovation hotspots in the food industry are centered on functional foods and advanced processing technologies.

Corporate applicants dominate the Top-1000 list, with all Top-10 patent owners being enterprises. This aligns with the entropy-based weighting results: firms are more likely than universities to engage in market-oriented behaviors such as PCT filing and patent transfer, which are strongly associated with high commercial value.

#### 4.5.2. Topic Mining via BERTopic

**Figure 8:**
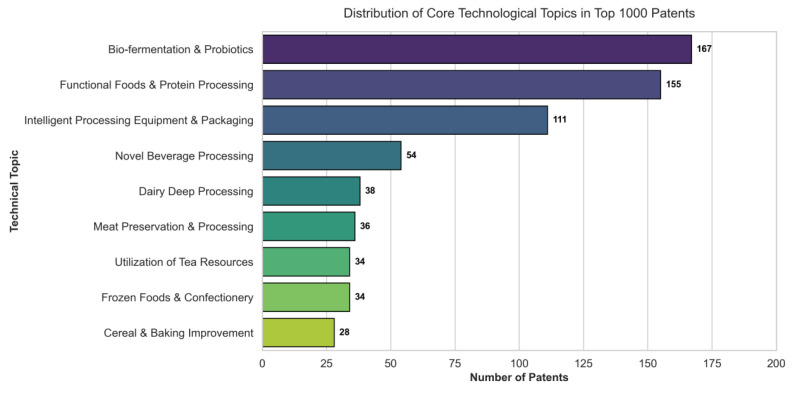
Distribution of Core Technological Topics among the Top 1000 High-Value Patents Identified via BERTopic.

To identify the dominant technological themes among “hidden champions”—high-scoring but non-awarded patents—BERTopic was applied. Nine stable topic clusters were identified, reflecting three major industry trends (see Figure 8).

First, bio-manufacturing and nutritional health emerged as the primary value highland. The top two clusters, “Bio-fermentation & Probiotics” (167 patents) and “Functional Foods & Protein Processing” (155 patents), collectively account for nearly 50% of the analyzed sample. This reflects a strategic industry shift towards synthetic biology and high-value-added ingredients, focusing on gut health modulation and plant-based protein innovations.

Second, intelligent manufacturing was identified as a critical driver of competitiveness. The cluster “Intelligent Processing Equipment & Packaging” (111 patents) ranked third, with keywords highlighting the industry’s rapid transition toward digitalized production lines, automated inspection systems, and smart packaging solutions.

Third, traditional sectors exhibited a clear trend of “Refined Upgrading.” Clusters such as “Dairy Deep Processing,” “Meat Preservation & Processing,” and “Utilization of Tea Resources” demonstrate that traditional food processing is evolving from simple manufacturing to technology-intensive deep processing, characterizing innovations in component separation, texture modulation, and waste resource utilization.

In summary, these findings confirm the proposed entropy model’s capacity to discover future-oriented core assets by precisely pinpointing the industry’s cutting-edge frontiers—namely Bio-tech, Intelligent Equipment, and Deep Processing—which represent the primary channels for the high-quality development of the future food industry.

## 5. Discussion

### 5.1. The Screening Mechanism of “Costly Signals” in Patent Value Formation

A key empirical finding of this study is that the entropy-based model assigns exceptionally high weights to PCT Application and Patent Transfer, together accounting for nearly half of the total weight. This result diverges considerably from traditional expert-based valuation systems, which typically emphasize technological indicators such as citation impact or technical breadth.

Interpreted through the lens of signaling theory, this outcome reveals an important value-screening mechanism in the food industry. The food industry is characterized by a high volume of patents relating to recipes, formulations, and process optimization. This abundance, combined with technological homogeneity, lowers the signal-to-noise ratio of text-based metrics such as claims, descriptions, or document length.

In contrast, PCT filings and market transfer represent “Costly Signals”, strategic actions requiring substantial financial commitment and legal planning. These actions therefore convey credible information about the applicant’s expectations regarding commercial potential. The entropy model captures this logic quantitatively. Because such costly actions occur infrequently, they exhibit high statistical dispersion (low entropy) and thus generate high information gain. This implies that in a market environment characterized by information asymmetry and technological convergence, commercial and legal behavior signals outperform purely technical signals in screening for core assets.

### 5.2. Divergence Between Objective Market Signals and Expert Review Criteria

Although awarded patents show significantly higher model scores on average, only five of them appear within the Top-100 high-value patent list generated by the model. This phenomenon highlights an important dimensional divergence between entropy-based market-oriented valuation and expert-based award evaluation.The China Patent Award emphasizes multiple dimensions, including technological novelty, social benefit, and alignment with national development priorities. These criteria reflect a broader policy and societal orientation rather than purely commercial considerations.

To address the potential limitation of using a single benchmark, it is crucial to distinguish between different forms of value recognition. The China Patent Award primarily serves as a proxy for “Industry Recognition” and “Social Recognition”, representing expert consensus and policy alignment. However, relying solely on this benchmark would indeed be one-sided for the food industry, which is highly profit-driven. Therefore, our proposed framework complements this by acting as a specialized proxy for “Market Recognition”.

In contrast, the entropy–TOPSIS model developed in this study is driven by data dispersion and signal scarcity, placing greater weight on commercial activity and legal enforcement intensity. As a result, the model identifies a set of high-scoring patents, many originating from multinational corporations or industry-leading enterprises, that exhibit strong market layout, frequent licensing, or active transfer behavior but have not participated in award application procedures. These patents can be regarded as “hidden champions”, technologically capable but more strongly distinguished by their commercial execution capacity. This divergence demonstrates that expert evaluation and entropy-based assessment are complementary rather than substitutive. Expert review incorporates normative and social welfare perspectives, whereas the entropy model captures market-driven value formation. Together, they offer a more comprehensive understanding of patent value.

## 6. Conclusions and Policy Suggestions

### 6.1. Conclusions

This study addresses the challenges associated with subjectivity, single-dimensional evaluation bias, and heavy-tailed data distributions in patent valuation within the food industry. Based on a large-scale dataset of 100,648 invention patents, we construct an objective patent valuation framework that integrates Shannon’s information entropy with the TOPSIS method and incorporates a square-root transformation to enhance robustness. The framework enables the identification of core technological assets from noise-intensive patent environments.

First, this study establishes a transparent, data-driven valuation paradigm. Unlike traditional approaches that rely heavily on textual or citation-based indicators, the proposed framework constructs a multidimensional evaluation system spanning technological, legal, and market–economic attributes. The combination of entropy weighting and nonlinear transformation reduces the influence of extreme outliers while preserving ordinal information. This design eliminates subjective bias in weight assignment and enables scalable, automated evaluation suitable for large patent portfolios. Furthermore, cross-validation against the Weighted Sum Model (WSM) confirmed the methodological robustness and generalizability of the proposed framework.

Second, the results reveal a commercial and legal dominance in patent value formation within the food industry. PCT applications, patent transfers, and legal event occurrences jointly exceed 73% of the entropy-derived weights. From an information-theoretic perspective, these behaviors represent rare, high-cost market signals that carry strong discriminatory value. Bootstrapping stability tests verified that this dominant weight distribution is statistically stable and not an artifact of sampling bias. This finding revises the conventional “technology-centered” view by demonstrating that, in the food industry, asset operation capability and market signaling play decisive roles in differentiating high-value patents.

Third, the proposed model demonstrates exceptional signal-to-noise efficiency. Validation against the China Patent Award benchmark shows that awarded patents achieve mean scores 2.64 times higher than ordinary patents, with a statistically significant difference at the 0.1% level. Moreover, the enrichment factor of high-value patents reaches 91.5 in the Top-100 ranked subset and peaks at 183.1 in the Top-50, indicating that the model is highly effective in concentrating valuable patents from large datasets. These results confirm the practical utility of entropy-based valuation in discovering “hidden champions” that may not be captured through expert review alone.

### 6.2. Policy Suggestions

Based on the empirical findings regarding the dominance of commercial and legal signals in patent valuation, we propose the following strategic recommendations for different stakeholders.

First, government evaluation systems should shift from quantity-oriented incentives toward international competitiveness. At the government level, there is an urgent need to accelerate the optimization of evaluation standards to cultivate the global standing of the food industry. Given the decisive role of PCT applications in screening high-value patents identified in this study, policy incentives should shift their focus from subsidizing the sheer quantity of patent applications to supporting overseas layout and technology transfer. Specifically, it is recommended to establish special funds dedicated to supporting the global patent layout of food enterprises, thereby enhancing the industry’s international discourse. Simultaneously, utilizing the objective entropy model proposed in this study, policymakers should establish a dynamic value-grading database. Patents evaluated as “High-Value” based on commercial signal strength should be granted policy privileges such as priority examination, fee reductions, or green channels for pledge financing. This approach effectively guides resources to congregate towards core assets through differentiated policy supply.

Second, enterprises should transition from patent accumulation to active asset operation. For enterprises, shifting focus from “controlling quantity” to “improving quality” is essential. Research indicates that mere technological accumulation is insufficient to validate market value; to distinguish their core assets in a homogenized market, firms must actively release “costly signals.” This necessitates conducting PCT international layouts for core technologies and activating “sleeping patents” through licensing and transfers, which releases strong value signals to the market to increase intangible asset valuation. Furthermore, considering the high weight assigned to legal events in the entropy model, enterprises should strengthen full-lifecycle legal maintenance. Actively engaging in patent pledge financing and licensing filing will enhance the legal stability and market visibility of their intangible assets, building a solid intellectual property moat.

Third, universities and research institutes must bridge the marketization gap through professional operation mechanisms. Universities and research institutes must address the significant mismatch between high technical scores and low market performance revealed by descriptive statistics. To bridge this “marketization gap”, academic institutions should move beyond the misconception that technology alone equates to value. It is recommended to establish professional patent operation agencies to facilitate the commercial conversion of academic achievements. Additionally, implementing a “Pre-Application Assessment” system based on the indicators constructed in this model is advised. By evaluating the commercial and international potential before filing, institutions can filter out low-value applications at the source. This prudent approach ensures that resources are concentrated on technologies with genuine market prospects, thereby improving the overall quality and maintenance rate of academic patent portfolios.

### 6.3. Limitations and Future Research

Despite its contributions, this study has several limitations that open avenues for future research. First, the use of the China Patent Award as the primary validation benchmark may not fully capture direct commercial success. Future research should incorporate alternative benchmarks such as licensing revenue or product-level sales data. Second, the current system lacks semantic analysis, suggesting that future work could integrate transformer-based language models to capture deeper technological nuances. Third, comparative studies across other technology-intensive sectors are needed to validate the universality of the “costly signal” mechanism beyond the food industry. Finally, extending the framework from ex-post evaluation to predictive assessment would further enhance its strategic relevance for decision-making.

## Figures and Tables

**Table 1:** Descriptive statistics of Awarded vs. Ordinary patents (Awd represents Awarded and Ord represents Ordinary).

Dimension	Indicator	Mean		Median		Std. Dev.		Multiple of
		Awd	Ord	Awd	Ord	Awd	Ord	Difference
Technology	Claims Count	6.07	5.9	5	6	4.13	3.4	1.03
	Examination Duration	2.51	1.63	2.16	0.56	1.09	2.05	1.54
	IPC Count	3.91	3.32	3	3	3.02	2.37	1.18
Legal Event Flag	Legal Event Flag	0.51	0.1	1	0	0.5	0.3	5.1
	PCT Flag	0.09	0.03	0	0	0.29	0.17	3
Market/Economic	Forward Citations	1.53	1.21	0	0	2.74	2.21	1.26
	Transfer Flag	0.18	0.06	0	0	0.39	0.23	3
	Applicants Count	4.55	2.65	4	2	2.89	2.15	1.72
	Emerging Industry Flag	0.96	0.84	1	1	0.19	0.37	1.14

**Table 2:** Performance comparison of different evaluation models.

Model	Weighting Method	Outlier Handling	Differentiation Ratio	EF_100_	Statistical Significance
Model I (Baseline)	Equal Weights	Square Root Transformation	1.60	18.3	1.05 × 10^−19^
Model II (Ablation)	Equal Method	None (Raw Data)	2.93	91.5	4.42 × 10^−18^
Model III (Winsorization)	Equal Method	Winsorization	2.20	91.5	6.43 × 10^−16^
Model IV (Proposed)	Equal Method	Square Root Transformation	2.64	91.5	9.73 × 10^−18^

Note: Differentiation Ratio is calculated as the mean score of the Awarded Group divided by the Ordinary Group. Enrichment Factor represents the ratio of the density of awarded patents in the Top 100 to their natural prevalence in the dataset. All *p*-values (<0.001) indicate statistical significance at the 99% confidence level.

## Data Availability

The original contributions presented in this study are included in the article. Further inquiries can be directed to the corresponding authors.
